# A gut microbiota-based predictive model for the treatment efficacy of Parkinson’s disease

**DOI:** 10.3389/fneur.2025.1686882

**Published:** 2025-12-18

**Authors:** Chen Liu, Tianxia Yu

**Affiliations:** Department of Neurology, Yantai Yantaishan Hospital, Yantai, China

**Keywords:** gut microbiota, Parkinson’s disease, fecal calprotectin, fecal lactoferrin, *E. coli*/Lactobacillus ratio

## Abstract

**Objective:**

This study aimed to develop and validate a predictive model for the decline in treatment efficacy among Parkinson’s disease (PD) patients based on clinical characteristics and biological markers, providing a basis for early risk identification and personalized therapeutic strategies.

**Methods:**

A retrospective study was conducted on 500 PD patients admitted to our hospital between January 2021 and December 2024. The patients were randomly divided into a training set (*n* = 350) and a validation set (*n* = 150) at a 7:3 ratio. Demographic characteristics, clinical rating scales, and biological markers were collected for all patients. In the training set, univariate analysis was performed to screen variables associated with treatment efficacy decline. After variable selection using LASSO regression, multivariate logistic regression analysis was performed to identify independent predictors. Predictive models, including random forest (RF), support vector machine (SVM), and gradient boosting, were constructed using Python 3.8.5 and the scikit-learn library. Model performance was evaluated using the area under the receiver operating characteristic curve (AUC), and the optimal model was selected based on key predictor importance.

**Results:**

No significant differences in baseline characteristics were observed between the training and validation sets (all *p* > 0.05). A multivariate logistic regression analysis identified the total MDS-UPDRS score, MDS-UPDRS II (activities of daily living), MDS-UPDRS IV (motor complications), PDQ-39 score, *E. coli*/Lactobacillus ratio, fecal lactoferrin, and fecal calprotectin as independent risk factors (all *p* < 0.05), while total fecal bacterial count was an independent protective factor (all *p* < 0.05). The RF model demonstrated superior predictive performance (AUC = 0.874, 95%CI: 0.831–0.917) compared to SVM (AUC = 0.806, 95%CI: 0.753–0.859) and gradient boosting (AUC = 0.842, 95%CI: 0.794–0.889).

**Conclusion:**

The RF model incorporating clinical and biological markers effectively predicts decline in treatment efficacy among PD patients, with fecal calprotectin, fecal lactoferrin, and the *E. coli*/Lactobacillus ratio serving as key predictors.

## Introduction

Parkinson’s disease (PD) is a prevalent neurodegenerative disorder characterized by motor symptoms, including bradykinesia, tremor, and rigidity, as well as various non-motor symptoms that diminish quality of life and significantly impair patients’ survival (1). Current clinical management primarily relies on pharmacotherapy, including levodopa; however, the majority of patients eventually fail to maintain a stable therapeutic response and develop wearing-off phenomena, characterized by symptom fluctuations and motor complications, which pose a major challenge in disease management (2, 3). Conventional efficacy assessments, based on clinical observations and rating scales, are limited by subjectivity and delayed detection, hindering early prediction of therapeutic response trajectories.

Recent studies indicate that wearing-off in PD is closely associated with gut microbiota dysbiosis, chronic inflammatory, and the progression of non-motor symptoms (4). For instance, reduced Lactobacillus and overgrowth of *Escherichia coli* in feces may influence neuroinflammation and dopaminergic neuron function via the gut–brain axis, while elevated fecal inflammatory markers (e.g., lactoferrin and calprotectin) suggest intestinal barrier disruption and systemic inflammation, potentially exacerbating therapeutic decline (5, 6). However, single biomarkers exhibit limited predictive value, and constructing a robust wearing-off prediction model by integrating multidimensional indicators remains challenging.

Machine learning algorithms, with their capacity for multi-source data integration, have demonstrated significant advantages in disease prognosis and therapeutic prediction. This study aims to develop a predictive model for wearing-off in PD by combining clinical scales, quality-of-life metrics, and fecal biomarkers via machine learning, identifying key determinants to guide early intervention and therapeutic optimization.

## Materials and methods

### Study participants

A retrospective cohort of 500 PD patients (determined by power analysis with 80% power, *α* = 0.05, detecting a 15% group difference in treatment response rates) diagnosed at our neurology department between January 2021 and December 2024 was enrolled. The inclusion criteria were as follows: (1) diagnosis conforming to the Clinical Diagnostic Criteria for Parkinson’s Disease (7), (2) ≥ 6 months of levodopa-based therapy, (3) complete clinical and follow-up data, including pre- and post-treatment efficacy assessments, and (4) availability of fecal microbiota and inflammatory marker profiles. The exclusion criteria were as follows: (1) secondary parkinsonism or Parkinson-plus syndromes; (2) severe cardiac, hepatic, or renal dysfunction; (3) intestinal infections, malignancies, or other conditions affecting fecal biomarkers; and (4) lost to follow-up or incomplete data. The patients were randomly divided into a training set (*n* = 350) and a validation set (*n* = 150) at a ratio of 7:3. This study was approved by the hospital’s ethics committee. All patients signed informed consent forms.

### Data collection

The following data from the electronic medical record system and the laboratory database were collected. Demographics: age and sex; Rating Scales: Movement Disorder Society-sponsored Unified Parkinson’s Disease Rating Scale (MDS-UPDRS; total score and Parts I–IV assessing non-motor symptoms, activities of daily living, motor symptoms, and motor complications, respectively), Parkinson’s Disease Fatigue Scale (PFS) (8), Non-Motor Symptoms Scale (NMSS), 39-item Parkinson’s Disease Questionnaire (PDQ-39), Wexner Constipation Scale, Geriatric Depression Scale (GDS), Parkinson’s Anxiety Scale (PAS), Lille Apathy Rating Scale (LAS), Parkinson’s Disease Sleep Scale (PDSS), Montreal Cognitive Assessment (MoCA), and Bristol Stool Form Scale.

Biological Markers: Defecation frequency (times/week), total fecal bacterial count, *E. coli*/Lactobacillus ratio, fecal lactoferrin, and fecal calprotectin (all fecal samples were collected within 1 week after enrollment and before standardized levodopa treatment), C-reactive protein (CRP), interleukin-6 (IL-6), plasma neurofilament light chain (NfL), serum ferritin, transferrin saturation, tumor necrosis factor-*α* (TNF-α), interferon-*γ* (IFN-γ), serum superoxide dismutase (SOD), serum dopamine, and serum glutathione (GSH).

### Outcome definition

The Movement Disorder Society Consensus on Therapeutic Response in PD (2021) explicitly designated short-term (≤ 6 months) as the core window for observing therapeutic efficacy (9). The Chinese Guidelines for PD Treatment (4th edition) recommend regular follow-ups every 3–6 months to assess symptom progression, manage drug side effects, and adjust treatment plans. Thus, 6 months is a widely recognized initial screening window for therapeutic efficacy in clinical practice (10). Patients were stratified into two groups based on 6-month follow-up data:

*Stable-response group:* patients in the stable-response group met all of the following criteria at 6 months: (1) ≥ 10% reduction or ≤5% fluctuation in MDS-UPDRS Part III score versus baseline; (2) absence of motor complications (e.g., wearing-off and dyskinesias), confirmed independently by two neurologists with ≥5 years’ experience using MDS criteria; and (3) ≤ 10% increase in levodopa equivalent daily dose (LEDD).
*Suboptimal-response group:* patients in the suboptimal-response group met any of the following criteria: (1) ≥ 10% increase in MDS-UPDRS Part III score, (2) presence of motor complications (same confirmation protocol), and (3) ≥ 20% LEDD escalation to maintain symptom control.

### Statistical analysis

Analyses were performed using SPSS 26.0, Python 3.8.5, and R 4.2.3. Normally distributed continuous variables were expressed as mean ± SD and compared via t-tests; non-normal data were reported as median (IQR) and analyzed using the Mann–Whitney U-tests. Categorical variables were presented as counts (%) and compared using χ^2^ tests. In the training set, univariate screening followed by LASSO regression and multivariate logistic regression modeling identified independent predictors, and their odds ratios (ORs) and 95% confidence intervals (CIs) were calculated. Multicollinearity was assessed via the variance inflation factor (VIF) with a threshold of VIF < 5 prior to multivariate modeling. Random forest (RF), support vector machine (SVM), and gradient boosting models were built using Python 3.8.5 (scikit-learn). The receiver operating characteristic (ROC) curve was plotted, and the area under the curve (AUC) value was calculated. A *p*-value of < 0.05 was considered statistically significant.

## Results

### Baseline characteristics of training and validation sets

A total of 500 PD patients were included. The training set (*n* = 350) included 198 (56.73%) stable-response patients, and the validation set (*n* = 150) included 84 (56%) suboptimal-response patients. There was no significant difference in the baseline characteristics between the two sets (all *p* > 0.05) (Table 1).

**Table 1:** Baseline characteristics of training and validation cohorts.

Variable	Training set (*n* = 350)	Validation set (*n* = 150)	*t/χ^2^*	*P*
Age (years)	65.23 ± 8.45	64.89 ± 7.98	0.419	0.675
Sex (male/female)	201/149	88/62	0.066	0.797
Hoehn & Yahr stage (I/II/III/IV/V)	42/135/110/55/10	17/58/46/23/6	0.088	0.993
Medication status (On/Off)	298/52	125/25	0.264	0.608
LEDD (mg/day)	523.67 ± 125.34	518.90 ± 119.87	0.395	0.693
MDS-UPDRS (total score)	56.32 ± 15.67	55.89 ± 14.98	0.285	0.776
MDS-UPDRS I	12.45 ± 4.23	12.12 ± 3.98	0.814	0.416
MDS-UPDRS II	18.67 ± 5.34	18.23 ± 4.98	0.861	0.390
MDS-UPDRS III	20.34 ± 6.78	19.89 ± 6.23	0.697	0.486
MDS-UPDRS IV	4.86 ± 2.12	4.67 ± 1.98	0.936	0.340
PFS score	15.67 ± 5.23	15.23 ± 4.91	0.878	0.381
NMSS score	32.45 ± 10.34	31.89 ± 9.87	0.563	0.574
PDQ-39 score	45.67 ± 12.31	44.88 ± 11.85	0.665	0.506
Wexner constipation score	8.23 ± 3.12	8.03 ± 3.02	0.663	0.508
GDS score	7.67 ± 2.89	7.45 ± 2.74	0.749	0.454
PAS score	10.34 ± 3.66	10.12 ± 3.45	0.627	0.521
LAS score	12.59 ± 4.18	12.17 ± 3.83	1.005	0.292
PDSS score	142.34 ± 25.61	140.77 ± 24.75	0.633	0.527
MoCA score	26.32 ± 3.25	26.16 ± 3.11	0.511	0.610
Defecation frequency (times/week)	4.23 ± 1.29	4.08 ± 1.13	1.235	0.217
Total fecal bacteria (CFU/g)	6.91 ± 1.34	6.78 ± 1.23	1.018	0.309
*E. coli*/Lactobacillus ratio	1.89 ± 0.67	1.82 ± 0.59	1.109	0.268
CRP (mg/L)	8.20 ± 3.17	8.04 ± 2.88	0.531	0.596
IL-6 (pg/mL)	15.62 ± 5.44	15.26 ± 5.15	0.689	0.491
NfL (pg/mL)	25.34 ± 8.67	24.82 ± 8.21	0.624	0.533
Simplified stool form scale score	3.67 ± 1.23	3.56 ± 1.12	0.941	0.347
Serum ferritin (ng/mL)	235.33 ± 68.19	230.75 ± 65.47	0.696	0.487
Transferrin saturation (%)	32.45 ± 8.34	31.90 ± 8.08	0.682	0.496
TNF-α (pg/mL)	12.38 ± 4.16	12.14 ± 3.93	0.601	0.548
IFN-γ (pg/mL)	9.69 ± 3.32	9.54 ± 3.06	0.434	0.636
SOD activity (U/mL)	85.34 ± 15.67	84.89 ± 15.35	0.296	0.767
Fecal lactoferrin (μg/g)	5.67 ± 2.12	5.55 ± 2.02	0.588	0.557
Serum dopamine (ng/mL)	125.31 ± 35.56	123.80 ± 34.23	0.440	0.660
GSH (μmol/L)	4.86 ± 1.34	4.72 ± 1.20	1.104	0.270
Fecal calprotectin (μg/g)	128.64 ± 45.43	125.95 ± 42.11	0.620	0.536

### Univariate analysis of factors influencing the attenuation of treatment efficacy in Parkinson’s disease

In the training set, the univariate analysis showed that statistically significant differences were observed between the stable-response group and the efficacy-attenuation group in the following indicators: MDS-UPDRS, MDS-UPDRS I, MDS-UPDRS II, MDS-UPDRS III, MDS-UPDRS IV, NMSS, PDQ-39, PDSS, MoCA, total fecal bacterial count, *Escherichia coli/*Lactobacillus ratio, fecal lactoferrin, and fecal calprotectin (all *p* < 0.05) (Table 2).

**Table 2:** Univariate analysis of factors influencing the attenuation of therapeutic efficacy in Parkinson’s disease.

Variable	Stable-response group (*n* = 152)	Efficacy-attenuation group (*n* = 198)	*t/χ^2^*	*P*
Age (years)	65.01 ± 7.98	66.14 ± 8.76	1.243	0.215
Sex (male/female)	142/110	59/39	0.429	0.513
Hoehn & Yahr stage (I/II/III/IV/V)	17/68/40/21/4	25/67/70/24/6	5.086	0.166
Medication status (On/Off)	215/37	83/15	0.022	0.883
LEDD (mg/day)	520.33 ± 120.65	531.61 ± 130.29	0.829	0.408
MDS-UPDRS (total score)	53.39 ± 12.48	58.72 ± 13.46	3.789	0.001
MDS-UPDRS I	11.82 ± 3.89	12.95 ± 4.73	2.389	0.017
MDS-UPDRS II	17.85 ± 5.44	19.36 ± 5.81	2.477	0.014
MDS-UPDRS III	19.49 ± 5.88	21.03 ± 6.74	2.238	0.026
MDS-UPDRS IV	4.23 ± 2.02	5.38 ± 2.27	4.926	0.001
PFS score	15.24 ± 4.83	15.96 ± 5.32	1.306	0.193
NMSS score	30.77 ± 9.84	33.08 ± 11.29	2.005	0.046
PDQ-39 score	43.79 ± 11.24	48.30 ± 13.45	3.335	0.001
Wexner constipation score	8.05 ± 2.98	8.61 ± 3.41	1.608	0.109
GDS score	7.23 ± 2.79	7.73 ± 3.12	1.555	0.121
PAS score	9.95 ± 3.56	10.72 ± 4.44	1.749	0.081
LAS score	12.06 ± 4.11	12.68 ± 4.53	1.321	0.187
PDSS score	145.67 ± 24.34	138.05 ± 26.92	2.735	0.007
MoCA score	26.56 ± 3.02	25.89 ± 2.87	2.116	0.035
Defecation frequency (times/week)	4.34 ± 1.35	3.89 ± 1.22	1.814	0.071
Total fecal bacteria (CFU/g)	7.32 ± 1.58	6.47 ± 1.43	5.266	0.001
*E. coli*/Lactobacillus ratio	1.76 ± 0.52	2.15 ± 0.78	5.322	0.001
CRP (mg/L)	8.12 ± 2.98	8.56 ± 3.23	1.306	0.192
IL-6 (pg/mL)	15.09 ± 4.24	16.03 ± 5.30	1.600	0.111
NfL (pg/mL)	24.56 ± 7.89	26.11 ± 9.23	1.657	0.098
Simplified stool form scale score	3.78 ± 1.20	3.57 ± 1.18	1.638	0.102
Serum ferritin (ng/mL)	238.67 ± 65.34	229.89 ± 62.12	1.281	0.201
Transferrin saturation (%)	32.12 ± 7.98	31.56 ± 8.23	0.639	0.523
TNF-α (pg/mL)	11.94 ± 3.51	12.68 ± 4.10	1.770	0.078
IFN-γ (pg/mL)	9.63 ± 2.98	10.16 ± 3.45	1.510	0.134
SOD activity (U/mL)	85.56 ± 14.98	83.34 ± 15.67	1.339	0.182
Fecal lactoferrin (μg/g)	5.12 ± 1.97	6.56 ± 2.33	6.122	0.001
Serum dopamine (ng/mL)	126.35 ± 32.45	124.78 ± 28.67	0.437	0.663
GSH (μmol/L)	4.98 ± 1.23	4.76 ± 1.12	1.745	0.082
Fecal calprotectin (μg/g)	112.79 ± 35.88	146.78 ± 52.34	6.863	0.001

### Multivariate logistic regression analysis of factors influencing the decline in treatment efficacy for Parkinson’s disease

The post-treatment efficacy status of patients was set as the dependent variable (1 = suboptimal-response group, 0 = stable-response group). Variables demonstrating statistical significance in the univariate analysis were incorporated into LASSO regression for variable selection (Supplementary Table 1). The screening criterion lambda.1se was applied to identify variables, as illustrated in Supplementary Figures 1, 2.

**Figure 1:**
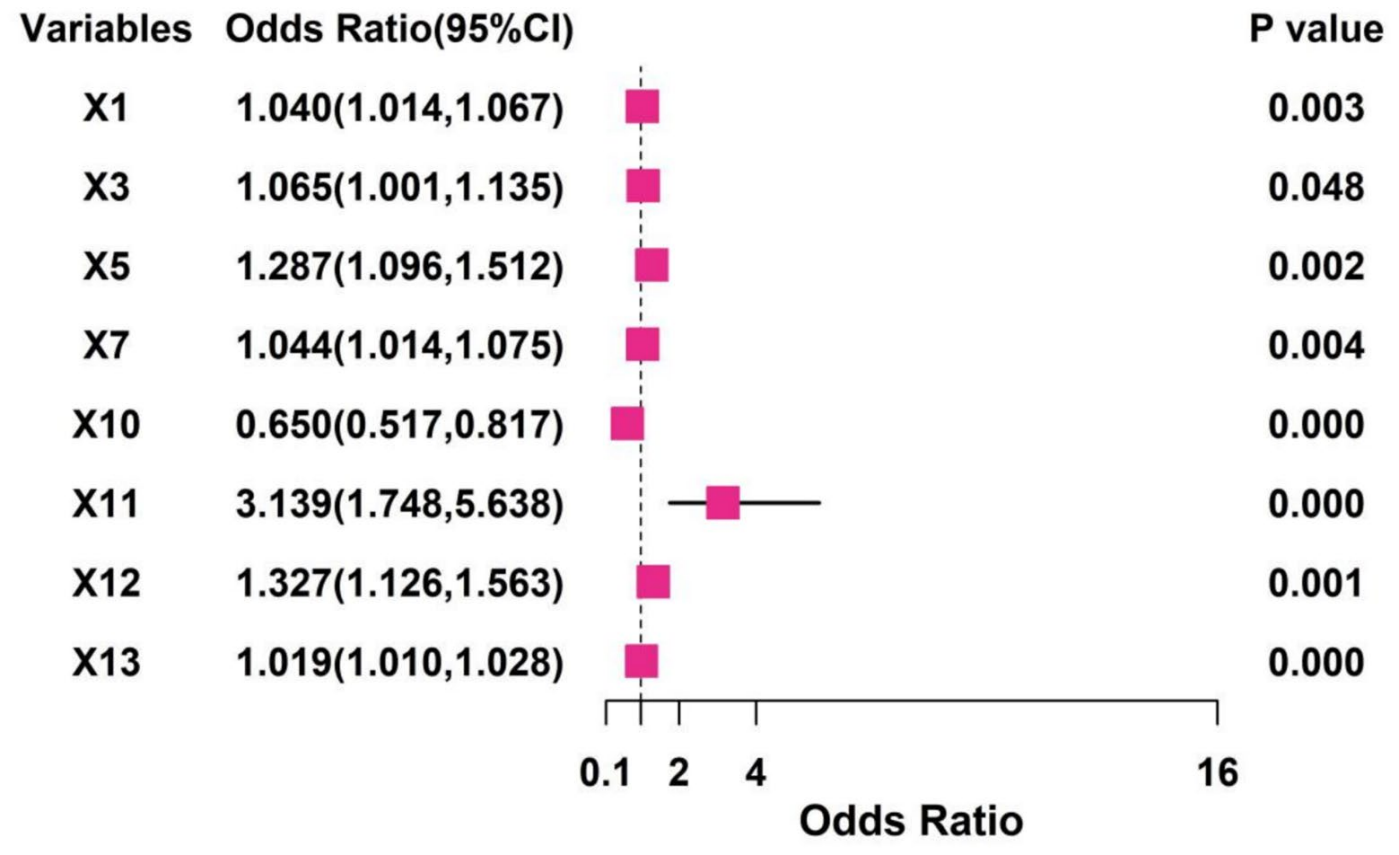
Results of the multivariate logistic regression analysis (X1: MDS-UPDRS, X3L: MDS-UPDRS II, X5: MDS-UPDRS IV, X7: PDQ-39, X10: total fecal bacterial count X11: *Escherichia coli*/Lactobacillus ratio, X12: fecal lactoferrin, X13: fecal calprotectin).

Variables with appropriate predictive capacity were subsequently included in the multivariate logistic regression analysis. The results revealed that MDS-UPDRS, MDS-UPDRS II, MDS-UPDRS IV, PDQ-39, *Escherichia coli*/Lactobacillus ratio, fecal lactoferrin, and fecal calprotectin were independent risk factors for the decline in treatment efficacy in Parkinson’s disease patients (all *p* < 0.05). Conversely, total fecal bacterial count was identified as an independent protective factor against treatment efficacy decline (all *p* < 0.05) (Figure 1).

### Prediction performance of machine learning models in training and validation sets

**Figure 2:**
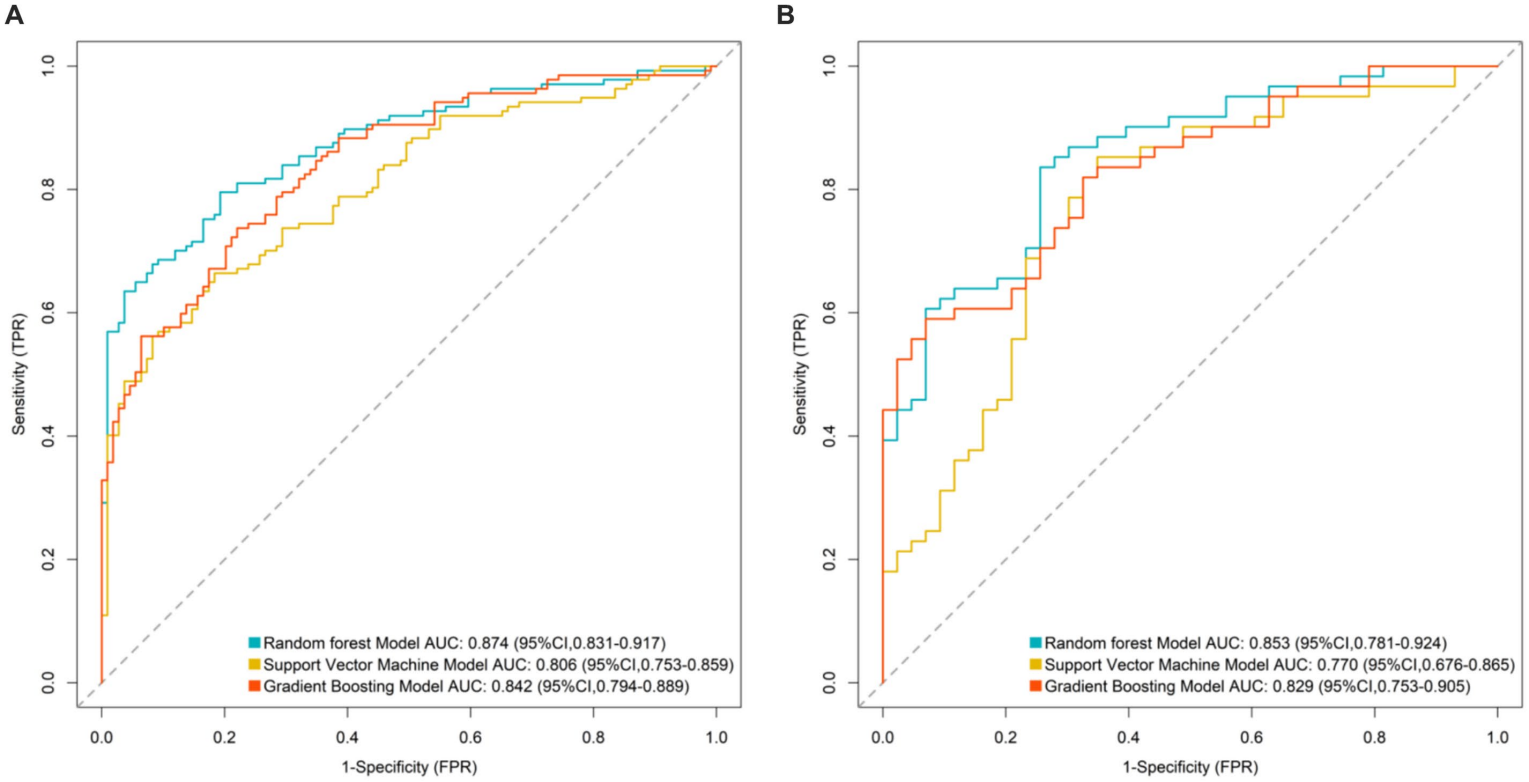
Area under the receiver operating characteristic curve of machine learning models (**A**: ROC curve for training set and **B**: ROC curve for validation set).

The random forest model, support vector machine model, and gradient boosting model were applied to both the training and validation sets. In the training set, the AUC values were 0.874 (95%CI: 0.831–0.917) for random forest, 0.806 (95%CI: 0.753–0.859) for support vector machine, and 0.842 (95%CI: 0.794–0.889) for gradient boosting. In the validation set, the corresponding AUC values were 0.832 (95%CI: 0.774–0.890), 0.768 (95%CI: 0.703–0.833), and 0.801 (95%CI: 0.738–0.864). The random forest model achieved the highest AUC values in both sets and was selected as the optimal model (Figure 2).

### Construction of the predictive model for diminished therapeutic efficacy in Parkinson’s disease

**Figure 3:**
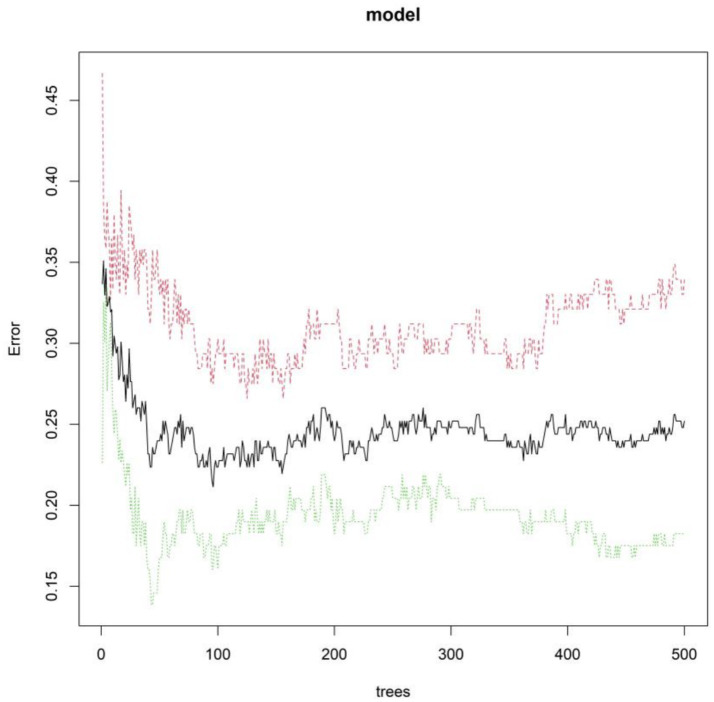
Trend of mean out-of-bag error rate with an increasing number of decision trees.

**Figure 4:**
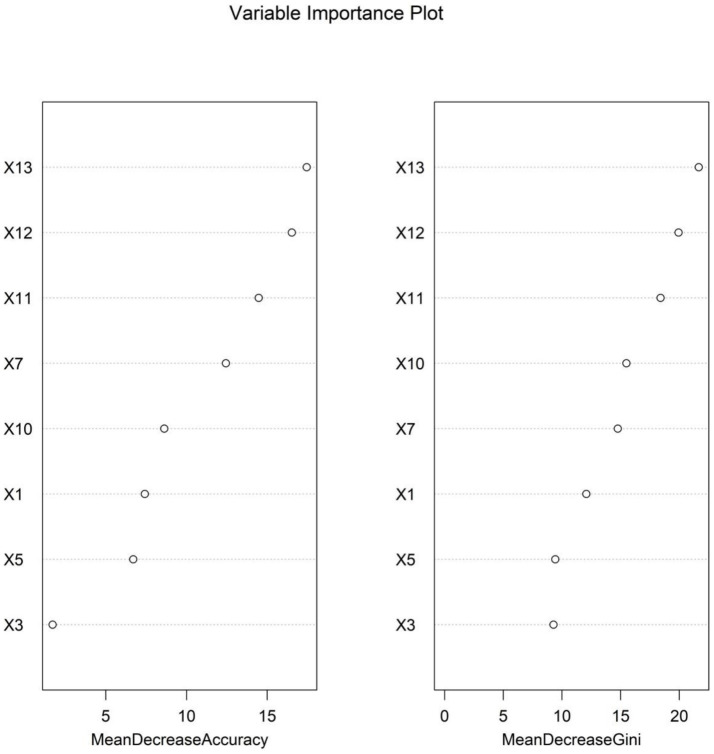
Variable importance ranking in the random forest model (note: X1: MDS-UPDRS, X3: MDS-UPDRS II, X5: MDS-UPDRS IV, X7: PDQ-39, X10: total fecal bacterial count, X11: *Escherichia coli*/Lactobacillus ratio, X12: fecal lactoferrin, and X13: fecal calprotectin).

The importance scores of independent influencing factors for diminished therapeutic efficacy in Parkinson’s disease were calculated using the random forest model. The descending order of feature importance was as follows: fecal calprotectin, fecal lactoferrin, *Escherichia coli*/Lactobacillus ratio, PDQ-39, total fecal bacterial count, MDS-UPDRS, MDS-UPDRS IV, and MDS-UPDRS II (Figures 3, 4).

## Discussion

The individual variability in therapeutic efficacy and the risk of efficacy decline during PD treatment remain core challenges in clinical management. Identifying the risk of efficacy decline early through objective indicators and optimizing treatment strategies are crucial for improving patients’ quality of life. This study integrated gut microbiota features, clinical rating scales, and inflammatory markers from 500 PD patients. Variables were screened using LASSO regression, and the multivariate logistic regression analysis identified the *Escherichia coli*/Lactobacillus ratio, fecal lactoferrin, and fecal calprotectin as independent risk factors for efficacy decline, while total bacterial count was an independent protective factor. A random forest model demonstrated the highest predictive performance (AUC = 0.874). Fecal calprotectin, lactoferrin, and the *E. coli*/Lactobacillus ratio ranked highest in feature importance, suggesting that gut microbiota characteristics and related inflammatory markers may serve as core biomarkers for predicting PD treatment efficacy. These findings provide a novel theoretical basis for precise clinical evaluation and personalized intervention strategies.

In this study, gut microbiota-related indicators played a pivotal role in predicting efficacy decline, which can be mechanistically explained by the multi-pathway regulatory network of the “gut-brain axis.” This involves the synergistic effects of gut microbial dysbiosis, intestinal barrier damage, and systemic inflammatory responses. An elevated *E. coli*/Lactobacillus ratio was a strong risk factor for efficacy decline (OR = 3.139), which was directly linked to disrupted gut–brain axis homeostasis due to microbial imbalance. Under normal physiological conditions, Lactobacillus, as a dominant species, maintains intestinal barrier integrity by secreting short-chain fatty acids (e.g., butyrate), suppresses pro-inflammatory cytokine release, and promotes the synthesis of neurotransmitter precursors such as *γ*-aminobutyric acid (GABA), thereby indirectly modulating central nervous system function (11, 12). In contrast, *E. coli* overgrowth may exacerbate therapeutic decline through multiple pathways, including the production of lipopolysaccharides (LPS), which activate intestinal mucosal immune cells (e.g., macrophages and dendritic cells) and trigger local inflammation (13). Additionally, *E. coli* disrupts tight junction proteins (e.g., occludin and claudin), thereby increasing intestinal permeability and allowing LPS and pro-inflammatory factors to enter systemic circulation, inducing low-grade systemic inflammation (14). Furthermore, *E. coli* may affect the substantia nigra via the vagus nerve or bloodstream, exacerbating oxidative stress damage in dopaminergic neurons and reducing drug sensitivity (15). The positive correlation between this ratio and MDS-UPDRS IV (motor complications) scores further supports the notion that microbial dysbiosis may accelerate efficacy decline by promoting motor complications.

Total bacterial count emerged as an independent protective factor (OR = 0.650), with higher levels indicating sufficient microbial abundance, reflecting the positive role of gut microbiota diversity in maintaining treatment response. A diverse gut microbiota is fundamental to metabolic homeostasis, with sufficient microbial abundance conferring multiple benefits (16). It enhances the synthesis of neuroactive compounds such as short-chain fatty acids and tryptophan metabolites, strengthening the suppression of central neuroinflammation (17). Additionally, it improves colonization resistance against pathogens, reducing the risk of intestinal infections and chronic inflammation (18). Moreover, sufficient microbial abundance helps stabilize the intestinal metabolism of drugs such as levodopa, regulating bioavailability and ensuring consistent therapeutic effects (19). Previous studies have confirmed that PD patients commonly exhibit reduced gut microbiota diversity, which correlates with disease severity. Our findings further suggest a positive association between microbial abundance and drug efficacy (20).

Fecal lactoferrin (a neutrophil activation marker) and calprotectin (a gut-specific inflammatory indicator) serve as direct evidence of intestinal barrier damage and immune activation. Both were independent risk factors for efficacy decline and ranked highest in feature importance in the random forest model, indicating that local intestinal inflammation may serve as a core predictive signal. The underlying mechanisms include the following: (1) gut inflammation spreading to the central nervous system via immune pathways of the gut–brain axis, activating microglia and promoting pro-inflammatory cytokine (e.g., IL-1β and TNF-*α*) release in the substantia nigra, thereby accelerating dopaminergic neuron degeneration (21); (2) intestinal motility dysfunction under inflammatory conditions, which affects drug absorption stability and leads to fluctuating plasma drug concentrations, thereby reducing therapeutic efficacy (22); and (3) chronic gut inflammation exacerbating neuroendocrine stress responses via the hypothalamic–pituitary–adrenal axis, thereby worsening motor fluctuations and drug tolerance (23). Notably, the correlation between these markers and MDS-UPDRS II (activities of daily living) scores suggests that gut inflammation may indirectly reduce treatment compliance by impairing daily functioning, creating a vicious cycle.

In this study, clinical indicators such as MDS-UPDRS total score, MDS-UPDRS II/IV, and PDQ-39 scores were incorporated into the optimal model alongside gut microbiota features, reflecting the multidimensional relationship between disease severity, functional status, and gut microbial ecology. For instance, MDS-UPDRS IV scores directly reflect motor complications (e.g., wearing-off and dyskinesia), which are not only dose-dependent but may also be accelerated by neuroinflammation triggered by microbial dysbiosis. The PDQ-39, a quality-of-life metric, positively correlated with fecal calprotectin levels, suggesting that gut inflammation may indirectly reduce subjective treatment satisfaction by exacerbating non-motor symptoms (e.g., pain and anxiety) (24, 25). The synergistic effects of these clinical and microbial indicators confirm the holistic regulatory role of the gut–brain axis in PD treatment response and highlight the limitations of relying solely on traditional scales, necessitating the integration of biomarkers for improved predictive accuracy.

The random forest model achieved a significantly higher AUC (0.874) than support vector machines and gradient boosting models, owing to its superior ability to integrate multidimensional data. It effectively captures non-linear relationships (e.g., interactions between the *E. coli*/Lactobacillus ratio and MDS-UPDRS III scores), reduces prediction variability caused by single-marker fluctuations through bootstrap sampling and decision-tree ensembles (particularly beneficial for fecal markers susceptible to detection variability), and provides intuitive feature importance rankings, highlighting the central role of gut inflammatory markers (calprotectin and lactoferrin) in guiding clinical monitoring. The model’s translational value lies in its potential to identify high-risk patients early via non-invasive fecal testing and simplified scale assessments, supporting personalized adjustments in drug dosing, probiotic supplementation (e.g., Lactobacillus), or anti-inflammatory interventions.

However, there are some limitations to this study. First, this single-center retrospective study may have selection bias due to the inherent limitations of retrospective data collection, which may affect the external validity of the findings. Second, the lack of external validation with multi-center datasets restricts the generalizability of the established random forest model to broader populations. Third, all fecal biomarkers were collected at a single time point (pre-treatment), which may not capture the dynamic changes of gut microbiota during PD progression; future studies should consider serial sampling to improve predictive accuracy. The 6-month follow-up period is relatively short considering the long-term progressive nature of PD, limiting our ability to assess the dynamic associations between gut microbiota and treatment efficacy over 3–5 years. Fourth, the use of probiotics or macrobiotics by patients was not systematically recorded in this study, which may act as confounding variables affecting gut microbiota composition and treatment response. Furthermore, gut microbiota analysis lacked metagenomic sequencing, potentially omitting key microbial features (e.g., species-specific metabolic pathways). The study did not explore gut microbiota modulation (e.g., probiotics) as an intervention, relying on indirect mechanistic evidence. Future multicenter prospective studies integrating metagenomics and metabolomics are needed to elucidate molecular pathways linking gut microbiota to drug efficacy, and external validation of the established random forest model will be conducted using multi-center datasets to enhance its clinical applicability. Subsequently, randomized controlled trials validating targeted interventions (e.g., probiotic supplementation) are warranted.

## Conclusion

Gut microbiota features are key predictors of PD treatment efficacy decline. The random forest model incorporating these markers demonstrated excellent predictive performance, with fecal calprotectin and lactoferrin serving as clinically accessible biomarkers. This study provides novel insights into optimizing PD treatment strategies from a gut–brain axis perspective, offering potential for improving therapeutic responses through targeted microbial modulation.

## Data Availability

The original contributions presented in the study are included in the article/Supplementary material, further inquiries can be directed to the corresponding author.
