# Supplementary material for: A gut microbiota-based predictive model for the treatment efficacy of Parkinson’s disease
Source: Front Neurol. 2025 Dec 18;16:1686882. doi: 10.3389/fneur.2025.1686882 (PMC12756069; doi:10.3389/fneur.2025.1686882)
Supplement: Supplementary file 1 [file Table_1.DOCX]

**Supplemental Table 1** Variable Assignment Table

| Variable | Indicator | Assignment |
| --- | --- | --- |
| X1 | MDS-UPDRS | Continuous variable |
| X2 | MDS-UPDRS I | Continuous variable |
| X3 | MDS-UPDRS II | Continuous variable |
| X4 | MDS-UPDRS III | Continuous variable |
| X5 | MDS-UPDRS IV | Continuous variable |
| X6 | NMSS | Continuous variable |
| X7 | PDQ-39 | Continuous variable |
| X8 | PDSS | Continuous variable |
| X9 | MoCA | Continuous variable |
| X10 | Total Fecal Bacterial Count | Continuous variable |
| X11 | E. coli/Lactobacillus Ratio | Continuous variable |
| X12 | Fecal Lactoferrin | Continuous variable |
| X13 | Fecal Calprotectin | Continuous variable |
| Y | Treatment Response Group | （1 = Suboptimal-response group，  0 = Stable-response group） |

**
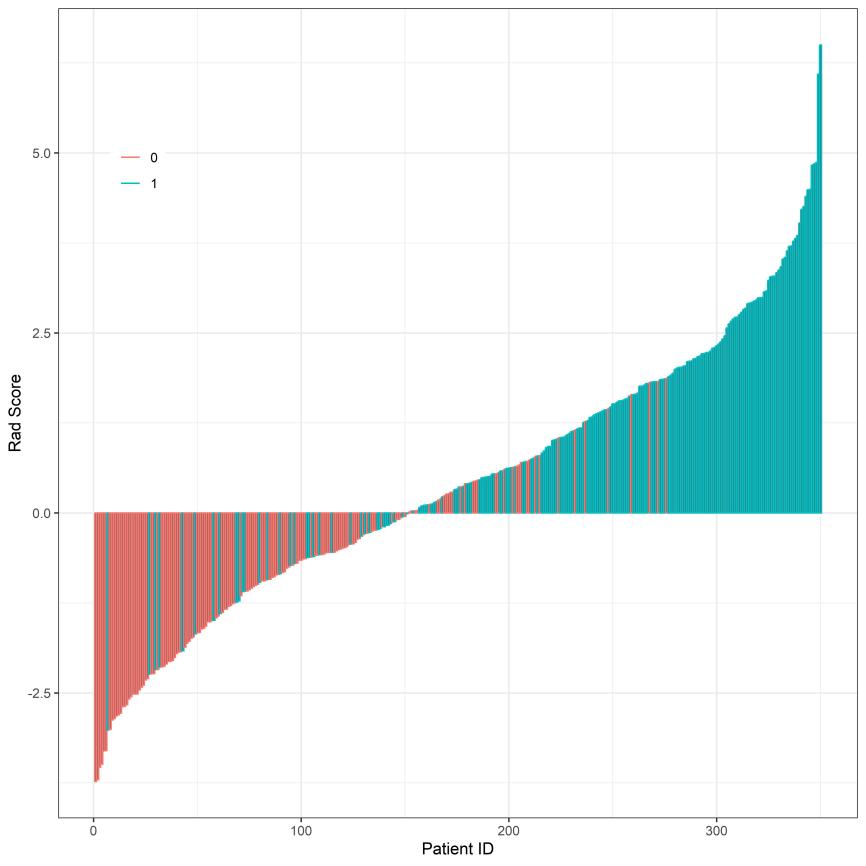
**

**Supplemental Figure 1** LASSO Rating Waterfall Chart


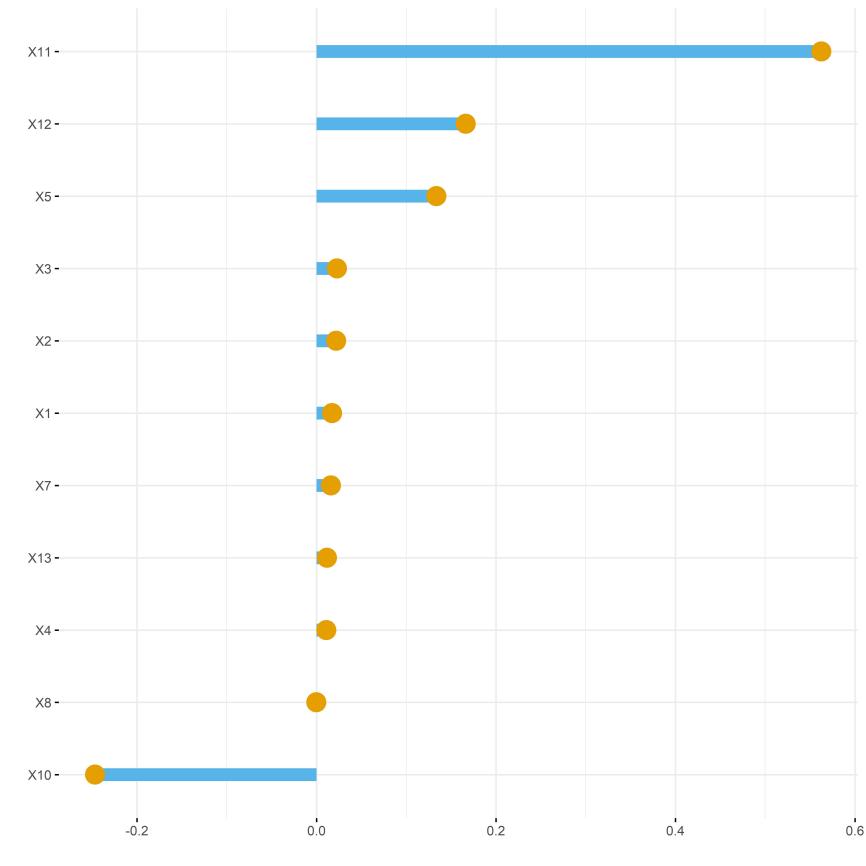


**Supplemental Figure 2**. Lamda.1se coefficient screening chart (Note: X1: MDS-UPDRS, X2: MDS-UPDRS I, X3: MDS-UPDRS II, X4: MDS-UPDRS III, X5: MDS-UPDRS IV, X6: NMSS, X7: PDQ-39, X8: PDSS, X9: MoCA, X10: Total fecal bacterial count X11: Escherichia coli/Lactobacillus ratio, X12: Fecal lactoferrin, X13: Fecal calprotectin)
